# Estimation of waste outflows for multiple product types in China from 2010–2050

**DOI:** 10.1038/s41597-021-00796-z

**Published:** 2021-01-18

**Authors:** Xianlai Zeng, Saleem H. Ali, Jinhui Li

**Affiliations:** 1 State Key Joint Laboratory of Environment Simulation and Pollution Control, School of Environment, Tsinghua University, Beijing, 100084 China; 2 College of Earth, Ocean and Environment, University of Delaware, Newark, DE 19709 USA; 3 Sustainable Minerals Institute, University of Queensland, Brisbane, Queensland 4072 Australia; 4 United Nations International Resource Panel, United Nations Environment Programme, Nairobi, Kenya

**Keywords:** Sustainability, Environmental impact

## Abstract

Material flow has been accelerated from underground natural minerals and is accumulating as aboveground waste stock. China is not only the largest producer and consumer of material-driven products, but also the largest generator of product waste. No official annual product waste data are released for China, which creates challenges especially in light of China’s emerging waste management policies. Previous studies have presented only estimations of waste streams for single products. In this study, we considered three product types and 33 technological products and collected all the available data. A Kuznets curve and Bass diffusion model were employed to forecast their future consumption. Based on urban consumption metabolism, we created one systematic estimation model of product waste generation related to material flow and social regulation. Typical technological product waste outflows were estimated from 2010 to 2050, which can assist further material flow and environmental impact research, as well as waste management policy-making and technology development. The created model can be potentially extended to other types of product waste estimation.

## Abstract (summary)

**Table:** 

Measurement(s)	waste generation • waste material
Technology Type(s)	digital curation • Mathematical Model
Factor Type(s)	year of data collection
Sample Characteristic - Location	China

Machine-accessible metadata file describing the reported data: 10.6084/m9.figshare.13277732

## Background & Summary

Materials derived from biotic, mined and secondary sources are fundamental to the functioning of modern technologically advanced society^1^. Since 1970, global material consumption in four main categories (e.g., metal ores, biomass, fossil fuels, and non-metallic mineral) has grown from 27 Pg (1 Pg = 10^15^ g) to 90 Pg in 2017^2^, and is projected to more than double from 79 Pg in 2011 to 167 Pg in 2060^3^. More and more underground materials are consumed in the technological product and finally “stored” in product waste. The rapid shortage of primary resources and the serious degradation of the environment from their extraction is one of the great challenges we face in transitioning to a more sustainable society^4,5^.

China’s rapid development has led to the growth of an unprecedented industrial metabolism in the past four decades. Driven by the national call for an “ecological civilization”^6^, China has embarked on an effort to implement solid waste management regulations and policies since 2010, particularly covering waste electrical & electric equipment regulation (WEEE), waste import ban, solid waste sorting, revised end-of-life vehicle (ELV) regulation, and zero-waste city. A major evolution of the environmental industry from informal to formal and from illegal to legal is emerging in the field of urban mining^7^ and circular economy^8^ paradigms. Lack of basic data related to typical technological product waste is severely frustrating the sound implementation of these regulations and policy.

National Development and Reform Commission regulated anthropogenic minerals for typical technological products waste, consisting of electrical & electric equipment (EEE), vehicle, and wiring & cable. Currently, China has no annual, officially released data of technological product waste generation. Only a few scholars previously attempted to forecast a certain type of product waste for a short duration; for instance, e-waste^9–11^ and ELV^12^. There is still no systematic estimation of solid waste generation, especially for typical technological product waste like WEEE, ELV, and waste wiring & cable (WWC). The chemical composition of materials stocks is scattered due to the fact that data collection tends to focus on single categories of materials. Therefore, after considering the consumption principle and the restrictive regulation, we firstly collected the available data of three types of technological product (i.e., EEE, vehicle, and wiring & cable) consumption from 1990s to now. Next, a Kuznets curve and Bass diffusion model will be employed to forecast their consumption from now to 2050. Finally, we provide the accurate estimation of product waste generation from the three technological product types and thirty categories from 2010 to 2050. Strict validation is also adopted with the detailed comparison and uncertainty analysis.

## Methods

**Fig. 1:**
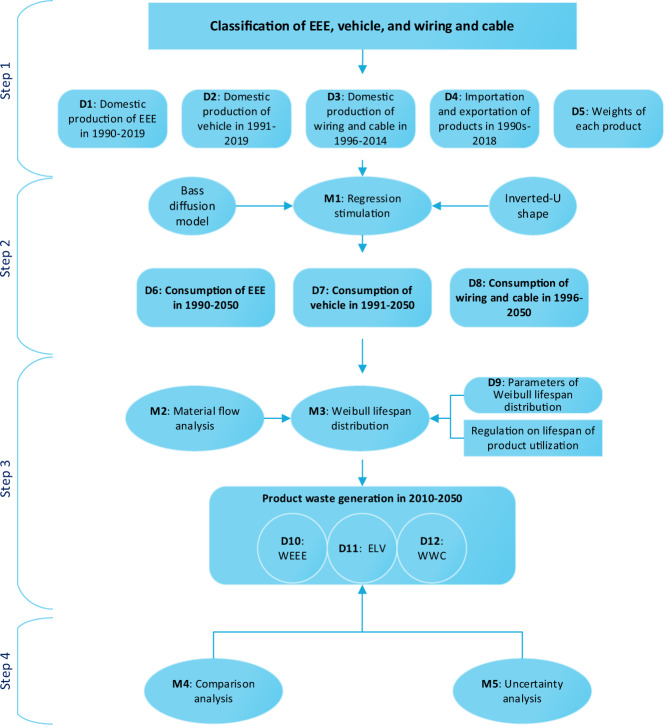
Research diagram of product waste estimation.

To estimate China’s technological product waste, we designed four steps combining: (1) data collecting and pre-mining for product consumption, (2) regression simulation for future consumption, (3) estimating the product waste generation, and (4) validating the obtained results. Figure 1 shows the detailed research diagram of product waste estimation with the datasets and methods which we employed for this study.

### Step 1. Data collecting and pre-mining for product consumption

**Fig. 2:**
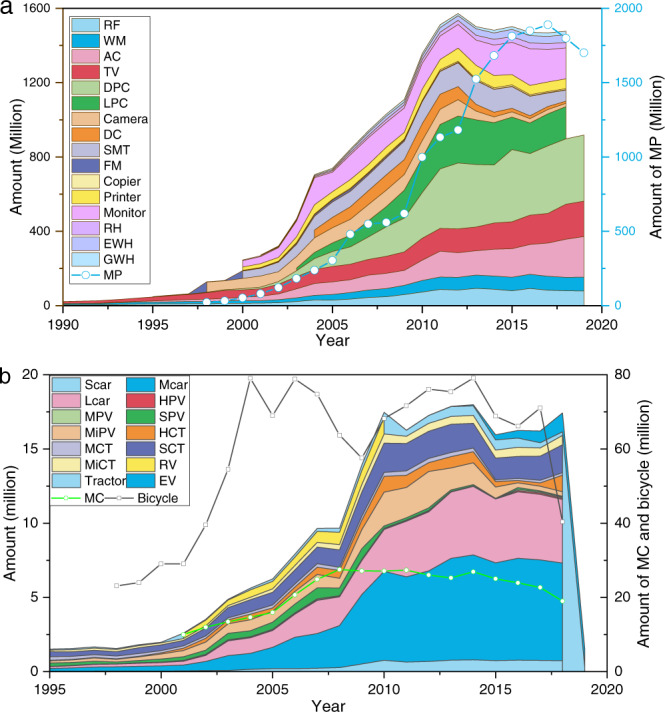
The domestic consumption of main products in China from 1990s to 2019. (**a**) EEEs; (**b**) Vehicle. Note: RF, refrigerator; WM, washing machine; TV, television; DC, Digital camera; SMT, single-machine telephone; FM, fax machine; RH, range hood; EWH, electric water heater; GWH, gas water heater; AC, air conditioner; DPC, desktop personal computer; LPC, laptop personal computer; MP, mobile phone; Scar, car with capacity ≤1 L; Mcar, car with Capacity: 1–1.6 L; Lcar, car with capacity >1.6 L; HPV: heavy passenger vehicle; MPV, middle passenger vehicle; SPV, small passenger vehicle; MiPV, mini passenger vehicle; HCT, heavy cargo truck; MCT, middle cargo truck; SCT, small cargo truck; MiCT, mini cargo truck; RV, refit vehicle; MC, motorcycle; EV, electric vehicle.

Based on the product classification of EEE, vehicle, and wiring & cable in China (Table 1), we collected all the available data of their production (Dataset, D1-D3 attached at figshare^13^), importation, and exportation (D4 attached at figshare^13^) from the 1990s to 2018/2019. All the data sources are primarily from China National Bureau of Statistics (http://data.stats.gov.cn/english/) and China Customs Statistics (http://www.tradedata.hk/). The domestic consumption amount in this period can be easily determined by the equation (Domestic consumption = Domestic production + Importation - Exportation). Seventeen types of EEEs and eight types of vehicles are involved here. Some products like mobile phones are still increasing, while a couple of products such as single-machine telephone and cathode-ray tube (CRT) monitors are dropping (Fig. 2).

**Table 1:** List of technical products considered in this study.

Type	Product	Type	Product
EEE	Refrigerator (RF)	Vehicle	Private passenger vehicle (PPV)
	Washing machine (WM)		Civil passenger vehicle (CPV)
	Air conditioner (AC)		New-registration passenger vehicle (NRPV)
	Television (TV)		Private cargo truck (PCT)
	Desktop personal computer (DPC)		Civil cargo truck (CCT)
	Laptop personal computer (LPC)		New-registration cargo truck (NRCT)
	Mobile phone (MP)		Car- Capacity ≤ 1 L
	Camera		Car- Capacity: 1–1.6 L
	Digital camera (DC)		Car- Capacity ≥ 1.6 L
	Single-machine telephone (SMT)		Refit vehicle (RV)
	Fax machine (FM)		Electric vehicle (EV)
	Copier		Motorcycle (MC)
	Printer		Tractor
	Monitor		Bicycle
	Range hood (RH)	Wiring & cable	Power cable
	Electric water-heater (EWH)		Telecommunication cable
	Gas water-heater (GWH)		Electromagnetic wire

### Step 2. Regression simulation for future consumption

In natural ecological systems, the inverted-U shape of a Kuznets curve is an adequate simulation model to address the evolution and succession of species, respectively^14,15^. This theoretical model has been well transplanted into industrial ecology to forecast the future product use^16–18^. But in reality, the consumption of technological products is dependent on total population, urbanization, and technology. China is projected to encounter the population peak in the 2020s^19,20^ and maintain a rapid urbanization in the 21^st^ century. At the micro level, the consumption from emergence to peak can be addressed appropriately by the Bass diffusion model^21,22^, which is innovatively considered for future consumption prediction in this study. Driven by emerging technologies, the dramatic decline or obsolescence of the consumption of one product is predominately subject to the substitution of another product^23^. For instance, the Cathode Ray Tube (CRT) has been replaced by liquid crystal display (LCD) so that the use of CRT has been sharply cut down^24,25^. Therefore, the rapid growth, stable growth, stagnation, and eventual decrease shown in the consumption model could be described approximately by exponential regression, linear regression, constant value, and linear regression, respectively.

With these principles, we deliberately drew the regression simulation equation based on the recent-year data (F1 attached at figshare^13^) and forecasted the future consumption of those products between the years of 2019/2020 and 2050. Accordingly, the domestic consumption of EEEs and vehicles from 1990 to 2050 is demonstrated in D6-D8 (see the detail at the below data code). Meanwhile, we also consider them to fit for normal distribution with standard deviations as 10% (of mean) between the years of 2019/2020–2030, 15% for 2030–2040, and 20% for 2040–2050. Additionally, we also create the weight dataset for all the concerning products (D5 attached at figshare^13^) based on the reported and self-determined data. The experienced evolution of the product weight appropriately encloses them as the Beta distribution^26^.

### Step 3. Estimating the technological product waste outflows

The Weibull distribution function is adequately sophisticated to express the durable product lifespan^27^. We use it to model the lifetime of the product. For no regulated-lifespan products like EEEs and bicycles, the probability density function (PDF) of the Weibull distribution is given by Eq. 1^10,28^. Regarding the regulated-lifespan products like vehicles, and wiring & cable, the regulated lifespan should be considered and embedded into the conventional PDF for Eq. 2.

1 $$f(t)=\left\{\begin{array}{cc}\frac{\beta }{\eta }{\left(\frac{t}{\eta }\right)}^{\beta -1}{e}^{-{(t/\eta )}^{\beta }} & (t\ge 0)\\ 0 & (t < 0)\end{array}\right..$$

2 $$f(t)=\{\begin{array}{cc}0 & (t > L)\\ {e}^{-{(t/\eta )}^{\beta }} & (t=L)\\ \frac{\beta }{\eta }{\left(\frac{t}{\eta }\right)}^{\beta -1}{e}^{-{(t/\eta )}^{\beta }} & (0\le t < L)\\ 0 & (t < 0)\end{array}.$$

where *β* is the shape parameter (*β* > 0), *η* is the scale parameter (*η* > 0), and *L* is the maximum lifetime of products regulated in China’s legislation system (y). EoL units for a particular duration time *t* can be mathematically described as ref. ^29^. Based on net product production (as inflow) and the lifespan function, the annual technological product waste generation (as outflow) can be determined by Eq. 3^30,31^:

3 $$\begin{array}{ccc}D(x) & = & \underset{0}{\overset{n}{\int }}f(x)P(x)dx\\  & = & \mathop{\sum }\limits_{i=1}^{30}[{P}_{i}(2000)\times {f}_{i}(x-2000)+{P}_{i}(2001)\times {f}_{i}(x-2001)+\cdots +{P}_{i}(x-1)\times {f}_{i}(1)].\end{array}$$

where *x* is the year; *D*(*x*) is the total weight of technological product waste generation in the year *x* (in Mg); *i* is the *i*^th^ category of product; *n* is the total number items in the product category; *P*_*i*_(20yy) is the net weight of the *i*^th^ product in the year 20yy (in Mg); and *f*_*i*_(x - 2000) is the obsolescence rate of the *i*^th^ product since the year 2000.

### Step 4. Validating the obtained results

Two approaches are enabled to validate the obtained results. Firstly, we compare the estimated technological product waste generation to the partially real data and previous studies. Secondly, based on all the equations, the uncertainty of the technological product waste generation and their materials stock is not only caused by the variables, including each product weight and Weibull function parameters, but also on the methods like the direct linear regression simulation. Here we consider their interactive influences and adopt a Monte Carlo simulation (10^5^ iterations) to examine the uncertainty of the obtained results.

## Data Records

After the deep data mining, we created the database containing a total of 5,478 data records (consumption and generation) for mainland China. Of these

- 922 are seventeen types of EEE consumption unit amount (from 1990 to 2050; D6 online at figshare^13^);
- 1,421 are twenty-four types of vehicle consumption unit amount (from 1990 to 2050; D7 online at figshare^13^);
- 320 are weight amount of six types of wiring & cable production, import, and export (from 1996 to 2050; D8 online at figshare^13^);
- 68 are the collected parameters of Weibull lifespan distribution and the regulated maximum lifetime (D9 online at figshare^13^);
- 697 are seventeen types of WEEE generation unit amount (from 2010 to 2050; D10 online at figshare^13^);
- 697 are seventeen types of WEEE generation weight amount (from 2010 to 2050; D10 online at figshare^13^);
- 615 are fifteen types of ELV generation unit amount (from 2010 to 2050; D11 online at figshare^13^);
- 615 are fifteen types of ELV generation weight amount from 2010 to 2050; D11 online at figshare^13^); and
- 123 are total three types of technological waste product generation weight amount (from 2010 to 2050; D12 online at figshare^13^).

**Fig. 3:**
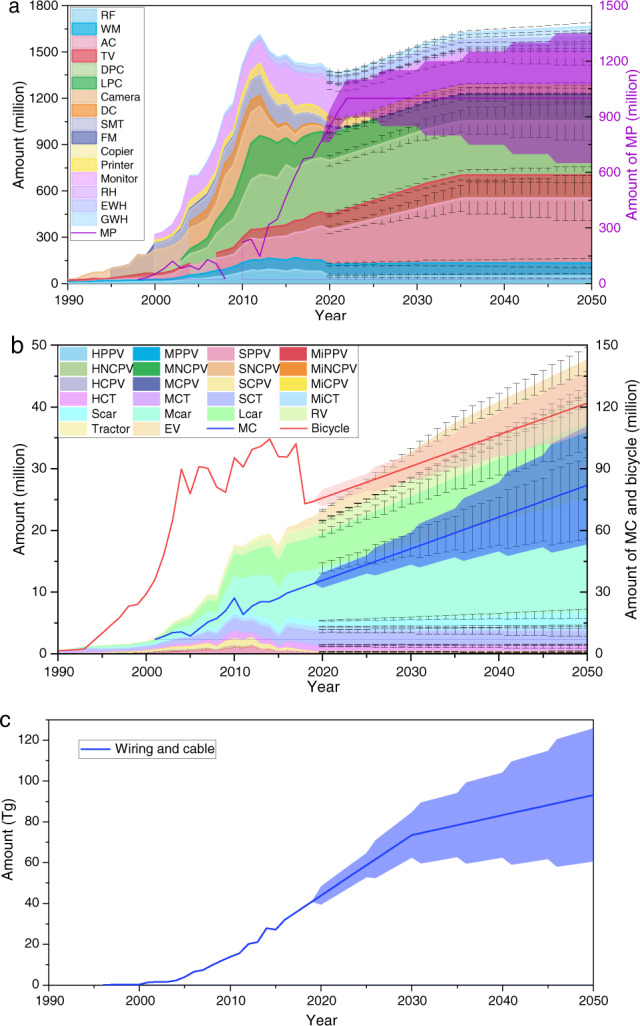
Projected consumption of typical products from 1990 to 2050: (**a**) EEE; (**b**) Main vehicle; (**c**) wiring & cable. Note: HPPV, heavy private passenger vehicle; MPPV, middle private passenger vehicle; SPPV, small private passenger vehicle; MiPPV, mini private passenger vehicle; HNCPV, heavy new-register civil passenger vehicle; MNCPV, middle new-register civil passenger vehicle; SNCPV, small new-register civil passenger vehicle; MiNCPV, mini new-register civil passenger vehicle; HCPV, heavy civil passenger vehicle; MCPV, middle civil passenger vehicle; SCPV, small civil passenger vehicle; MiCPV, mini civil passenger vehicle; HCT, heavy cargo truck; MCT, middle cargo truck; SCT, small cargo truck; MiCT, mini cargo truck; Scar, car with capacity ≤ 1 L; Mcar, car with Capacity: 1–1.6 L; Lcar, car with capacity >1.6 L; RV, refit vehicle; MC, motorcycle; EV, eletric vehicle. The error and shade area indicate the range of consumption.

China’s domestic consumption of EEE, vehicles, and wiring & cable from 2010 to 2050 is projected and demonstrated in Fig. 3. After 2030, the total amount of EEE consumption will reach the peak of 2500 million units. The total consumption of vehicles and wiring & cable, however, will grow constantly during this period of time.

**Fig. 4:**
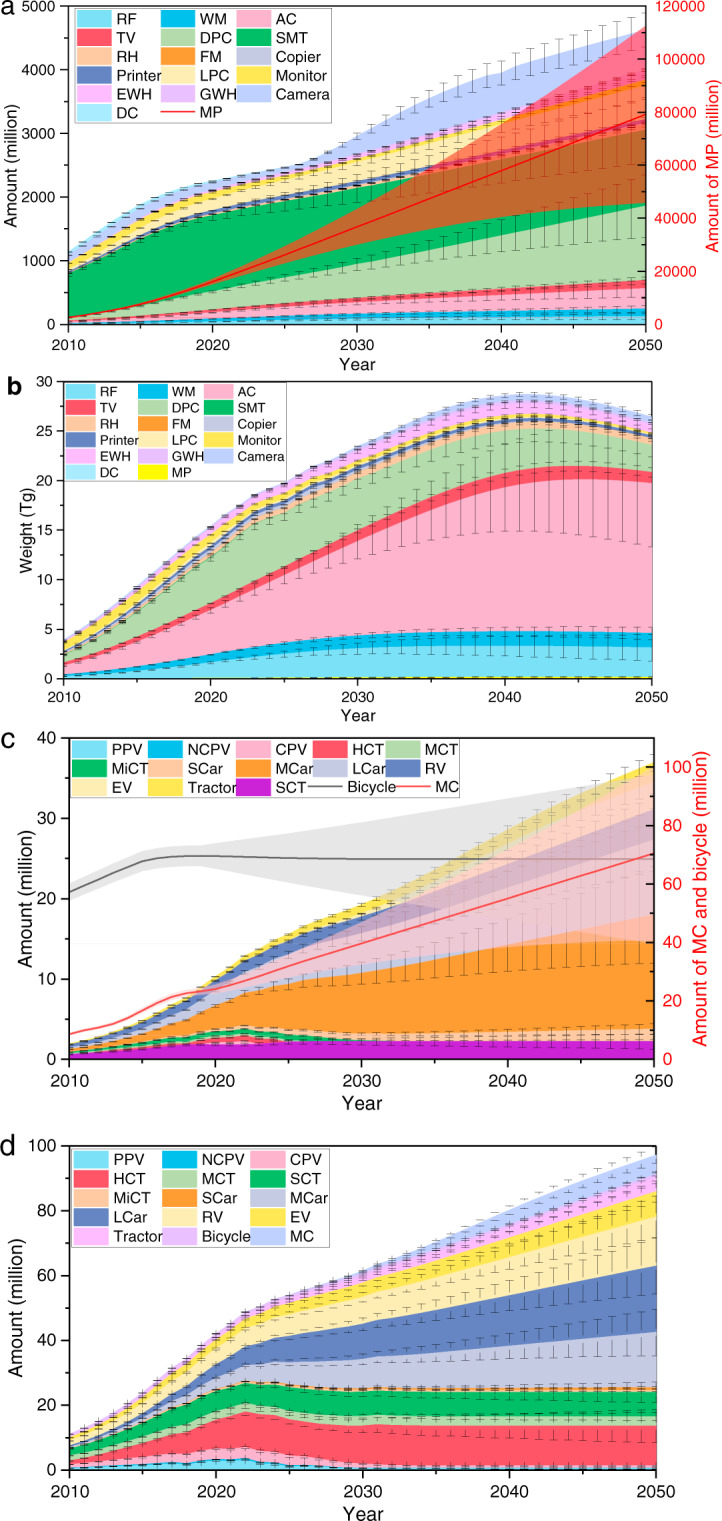
Weight of product waste outflows from 2010 to 2050: (**a**) unit amount of WEEE; (**b**) weight amount of WEEE; (**c**) unit amount of ELVs; (**d**) weight amount of ELVs. Note: PPV, private passenger vehicle; NCPV, new-register civil passenger vehicle; CPV, civil passenger vehicle; HCT, heavy cargo truck; MCT, middle cargo truck; SCT, small cargo truck; MiCT, mini cargo truck. The error and shade indicate the range of consumption.

We also visualize the evolution of various WEEE and ELVs outflows from 2010 to 2050 (Fig. 4). Regarding WEEE, the total unit and weight amount will grow from 635 million and 4 Tg (1Tg = 10^12^g) in 2010 to approximately 4,000 million and 28 Tg in 2040, respectively. The peak time is projected to be around 2040–2045 year. With respect to ELVs, the total unit and weight will maintain a rise driven by economic growth and rapid urbanization. The total weight amount will grow from 11 Tg in 2010 to 42 Tg in 2020 and 100 Tg in 2050.

The obtained data could be applied directly in a variety of fields. For instance, the estimated product waste generation can indicate the future recycling potential of a resource: how about the economic benefit and the ascending size of the waste recycling industry? Thus, potential energy conservation and associated greenhouse gas emission reduction can be enclosed from the recycling of product waste. Last but not least, the future consumption and obsolescence of electronics and vehicles will indicate a promising consumer electronics and automobile industry in a planned way^32^.

## Technological Validation

### Comparison with existing works

**Fig. 5:**
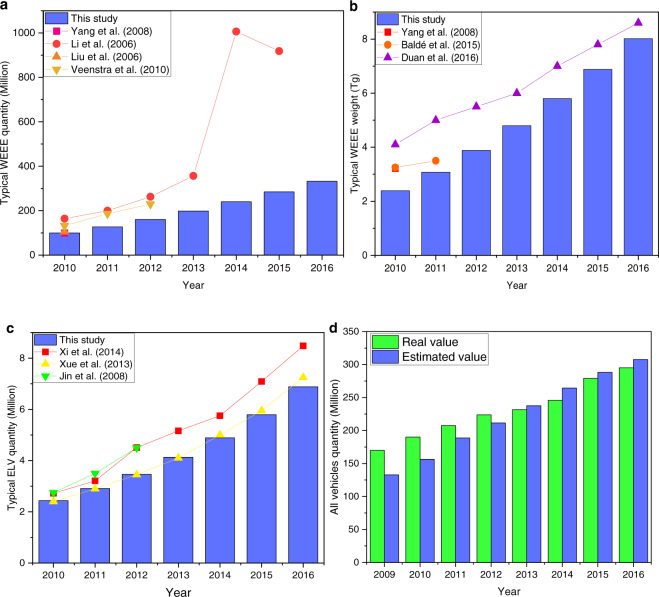
The comparison of WEEE and ELV studies: (**a**) Typical WEEE quantity; (**b**) Typical WEEE weight; (**c**) Typical ELV quantity; (**d**) All the registered vehicles quantity. Note: Typical WEEE covered RF, WM, TV, PC, and AC; typical ELV covered passenger vehicle, cargo truck, car, and refit vehicle. Yang *et al*.^35^, Li *et al*.^36^, Liu *et al*.^37^, Veenstra *et al*.^38^, Baldé *et al*.^9,17^, Duan *et al*.^10^, Xi *et al*.^33^, Xue *et al*.^39^, and Jin *et al*.^40^. Note: updated from Zeng *et al*.^32^, Copyright © 2020, Springer Nature.

We compared our obtained results with the existing research and real values. Regarding five typical types of WEEE (e.g., TV, air conditioner, refrigerator, washing machine, and personal computer), their total quantity from 2010–2016 from this estimation is somewhat lower than the values reported by others previous studies (Fig. 5). The difference can be attributed to two aspects of the previous works: old used data sources and different estimating methods. Moreover, in weight, the gaps among these studies are not significant. Regarding typical ELV quantity, some previous works indicate no distinct difference to this study. The biggest difference is given from Xi *et al*.^33^ only using a simple linear regression. The latest data until 2019 and the scientific methods such as Bass diffusion model and Weibull lifespan distribution were employed in this study to realize an accurate estimation. Additionally, the registered vehicle data has been estimated in this study for further comparison with real value released by the government. With the variance analysis, they demonstrate almost the same value without significant difference. The difference in 2009 is much larger than one in 2016 because the available consumption data of vehicle is initialed from 1995 (Fig. 2). The data in 2009 is more sensitive to the unknown information before 1995. All the discussions can verify and validate the above results of this study, and further consolidate the relevant results.

### Data error and uncertainty analysis

The error or range of the obtained results is primarily related to two points: one is the estimating waste methods of the Weibull distribution and the other is the projected future consumption of technological products. The error in the data of product waste outflows from 2010 to 2019 is provided from the Weibull distribution, but the error for the year of 2020 to 2050 is afforded by the two mixed points. Perhaps the projected future consumption method plays a bigger role.

**Fig. 6:**
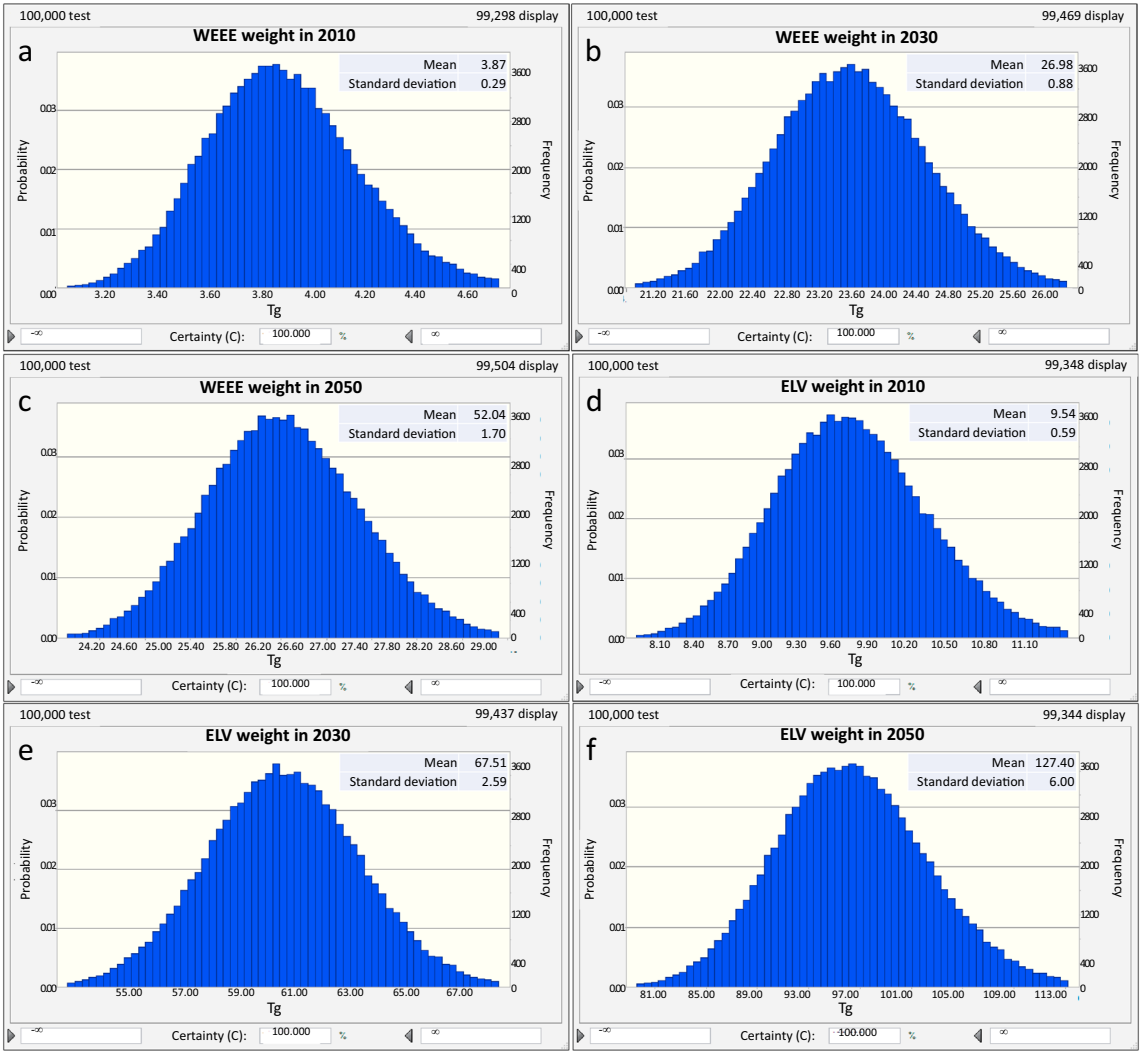
Uncertainty of waste product generation: (**a**) WEEE weight in 2010; (**b**) WEEE weight in 2030; (**c**) WEEE weight in 2050; (**d**) ELV weight in 2010; (**e**) ELV weight in 2030; (**f**) ELV weight in 2050.

We adopted the Monte Carlo simulations to further validate the obtained results. The uncertainty of waste product generation mainly depends on each product weight (Beta distribution) and the Weibull lifespan parameters (Normal distribution). Normal distribution was assumed for the forecasted data of EEE and vehicle consumption (see Online D6 and D7 at figshare^13^) with the standard deviation of 10%, 15%, and 20% for the data in 2020–2030, 2031–2040, and 2041–2050, respectively. We chose the WEEE weight and ELV weight in 2010, 2030, and 2050 as the examples. The increase of standard deviation indicates a growth in the uncertainty of waste product weight (Fig. 6). Nevertheless, all 100% certainties can fully verify the robustness of the waste product outflows.

## Data Availability

All relevant data (D1-D12) and figure (F1) presented in this article are available online at figshare^13^. They were developed using the collecting data and Eqs. (1–3) in detail described in the methods. Equation (1) was employed for EEE and wiring & cable to measure the waste outflows, and Eq. (2) was adopted for vehicle to measure the waste outflows. It should be highlighted here that some obtained data covers the year until the year of 2018 or 2019 (D1-D8), which has updated the given data until the year of 2015 or 2016 at the published article in Nature Communication^34^.
